# Dobrava-Belgrade Virus Spillover Infections, Germany

**DOI:** 10.3201/eid1512.090923

**Published:** 2009-12

**Authors:** Mathias Schlegel, Boris Klempa, Brita Auste, Margrit Bemmann, Jonas Schmidt-Chanasit, Thomas Büchner, Martin H. Groschup, Markus Meier, Anne Balkema-Buschmann, Hinrich Zoller, Detlev H. Krüger, Rainer G. Ulrich

**Affiliations:** Friedrich-Loeffler-Institut–Institute for Novel and Emerging Infectious Diseases, Greifswald-Insel Riems, Germany (M. Schlegel, T. Büchner, M.H. Groschup, A. Balkema-Buschmann, R.G. Ulrich); Charité-Universitätsmedizin Berlin, Berlin, Germany (B. Klempa, B. Auste, D.H. Krüger); Institute of Slovak Academy of Science, Bratislava, Slovakia (B. Klempa); Landesforstanstalt Mecklenburg-Vorpommern, Schwerin, Germany (M. Bemmann); Bernhard-Nocht-Institute for Tropical Medicine, Hamburg, Germany (J. Schmidt-Chanasit); University of Lübeck, Lübeck, Germany (M. Meier); University of Rostock, Rostock, Germany (H. Zoller); 1 These authors contributed equally to this article.

**Keywords:** Dobrava-Belgrade virus, hantavirus, vector-borne infections, rodent-borne pathogens, viruses, Germany, dispatch

## Abstract

We present the molecular identification of *Apodemus agrarius* (striped field mouse) as reservoir host of the Dobrava-Belgrade virus (DOBV) lineage DOBV-Aa in 3 federal states of Germany. Phylogenetic analyses provided evidence for multiple spillover of DOBV-Aa to *A. flavicollis,* a crucial prerequisite for host switch and genetic reassortment.

European hantaviruses are emerging viruses that can cause hemorrhagic fever with renal syndrome (HFRS) of differing severities. Dobrava-Belgrade virus (DOBV) is a hantavirus that appears in 3 distinct lineages hosted by different *Apodemus* species. The DOBV-Af lineage associated with the yellow-necked mouse (*A. flavicollis*) has caused serious HFRS in southeast Europe with a case-fatality rate <12% (*1*,*2*). Human infections with Caucasian wood mouse (*A. ponticus*)–associated DOBV-Ap have resulted in more moderate than severe HFRS in the southern part of European Russia (*3*). Mild-to-moderate human DOBV disease in central and eastern Europe has been connected with infection by DOBV-Aa lineage carried by the striped field mouse (*A. agrarius*) (*3*–*5*). Other *A. agarius*–associated strains, found in Estonia and called Saaremaa virus, have been proposed to form a distinct hantavirus species (*6*). In Germany, human DOBV cases with mild to moderate clinical outcomes have been detected by serologic investigations (*4*,*7*) but only 1 short DOBV-Aa small (S) segment sequence derived from a patient in northern Germany has been identified (*8*). The natural host and the geographic distribution of DOBV in its reservoir host has remained unknown in Germany.

## The Study

**Figure 1:**
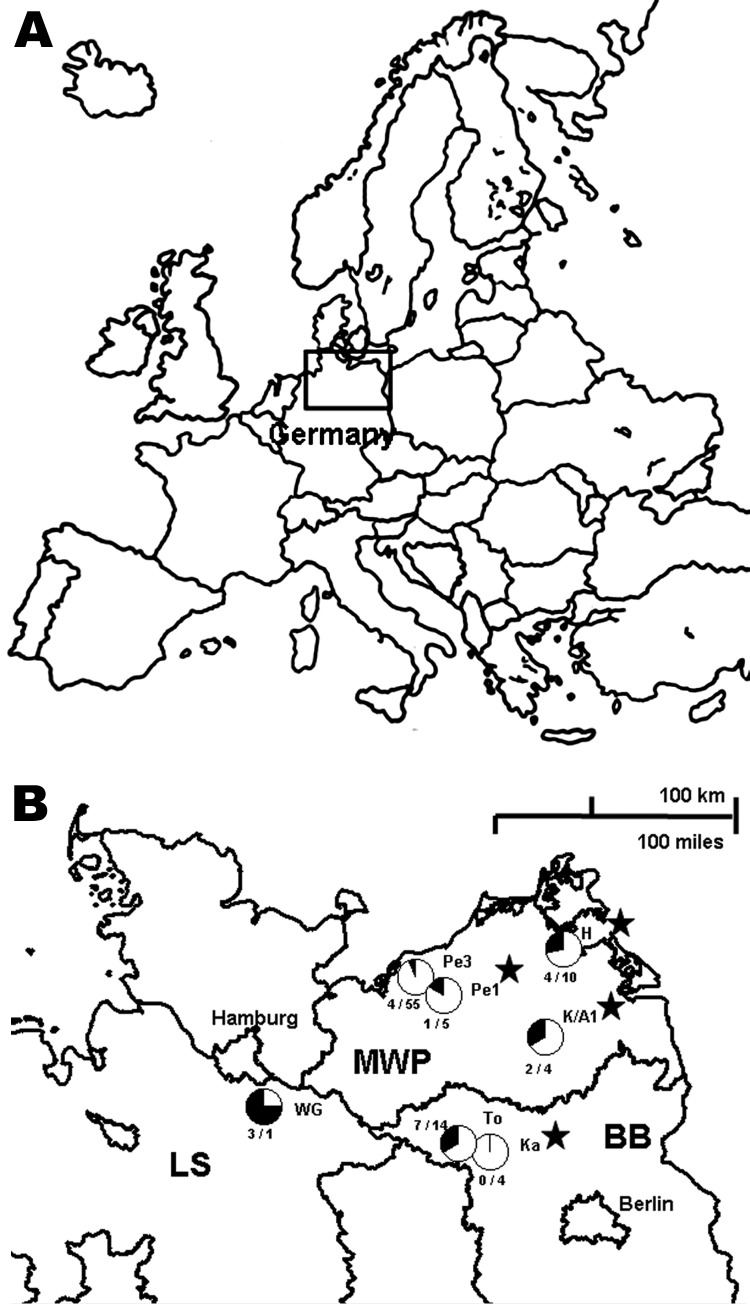
Seroprevalence of Dobrava-Belgrade virus (DOBV) in *Apodemus* *agrarius* mice within 3 federal states of Germany, central Europe. A) Location of the study area (box). B) Locations of the study sites. WG, Lüneburg district, Lower Saxony (LS); Pe1 and Pe3, Güstrow district; H, Nordvorpommern district, K/A1, Demmin district, all Mecklenburg-Western Pomerania (MWP); To and Ka, Ostprignitz-Ruppin district, Brandenburg (BB). For each trapping site, the rate of seroreactive *A. agrarius* mice is given as a circle (seroreactive and equivocal samples in black, negative samples in white) and with the numbers of seroreactive and equivocal samples/negative samples. At sites Pe1, H, K/A1, and Ka, 1 seroreactive *A. flavicollis* mouse was detected in each location (marked with stars). At site Pe1, 1 equivocal sample was found by the DOBV immunoglobulin (Ig) G ELISA. In addition to *Apodemus* mice, 138 rodents of other species, including 116 bank voles (*Myodes glareolus*), were trapped during the same period at sites Pe1, Pe3, K/A1, Ka, and To, but none of the 136 rodents with available transudates reacted in the DOBV IgG ELISA. Transudates with an optical density (OD) value below the lower cutoff (average of the OD values determined for 2 parallel tests of a negative *Apodemus* spp. serum control; average 0.041) were regarded as negative. Samples with an OD value above the upper cutoff (2-fold of the lower cutoff; average 0.082) were regarded as positive. Samples showing OD values between the lower and upper cutoffs were regarded as equivocal.

During 2002 through 2008, a total of 366 *Apodemus* mice were trapped at 7 different sites in Germany (Figure 1). Serologic screening of transudates collected from these rodents by using an in-house DOBV immunoglobulin (Ig) G-ELISA, with a yeast-expressed nucleocapsid protein of DOBV-Af as antigen, identified 16 reactive and 5 equivocal samples of 114 *A. agrarius* trapped at 7 trapping sites in 3 federal states of Germany (Figure 1; Technical Appendix). Additionally, of 237 *A. flavicollis* mice, 1 equivocal sample and 4 DOBV-reactive samples were detected at 4 trapping sites (Figure 1; Technical Appendix). In contrast, of 15 wood mice (*A. sylvaticus*) originating from 3 trapping sites, none were found to be DOBV-seroreactive. A subsequent focus-reduction neutralization test showed a higher endpoint titer with DOBV-Aa than DOBV-Af (Technical Appendix) for 6 of the 8 investigated transudates independently, whether originating from *A. agrarius* or *A. flavicollis*.

**Figure 2:**
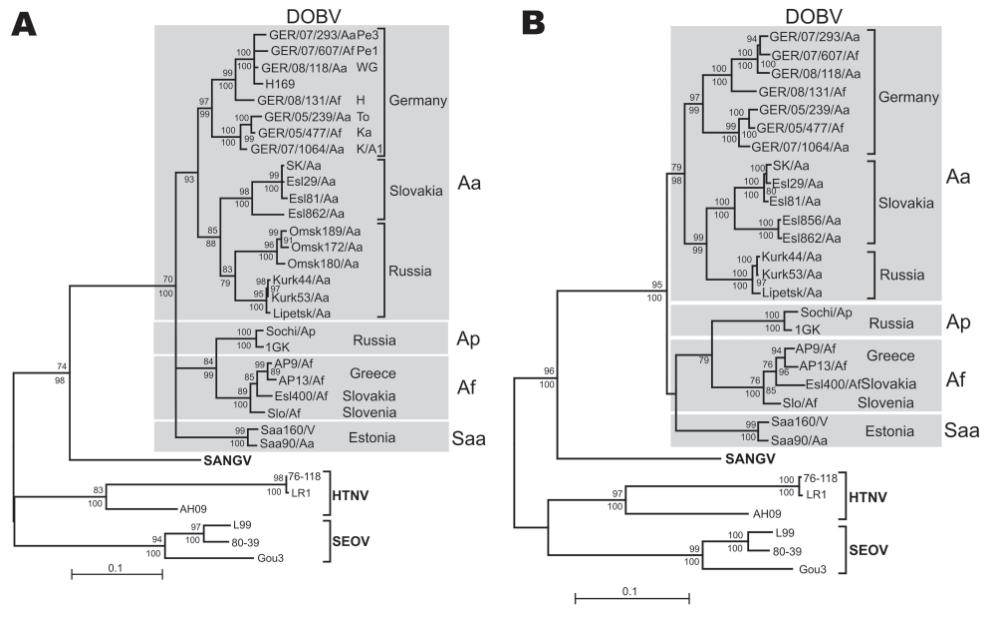
Maximum-likelihood (ML) phylogenetic trees of Dobrava-Belgrade virus (DOBV) based on partial small (S) segment nucleotide sequences of 559 nt (position 377–935) (A) and complete nucleocapsid protein coding nucleotide sequences (S segment open reading frame) (B). The ML trees (Tamura-Nei evolutionary model) were calculated using TREE-PUZZLE package (www.tree-puzzle.de). Scale bars indicate an evolutionary distance of 0.1 substitutions per position in the sequence. Values above the branches represent PUZZLE support values. Values below the branches are bootstrap values of the corresponding neighbor-joining tree (Tamura-Nei evolutionary model) calculated with the PAUP* software (paup.csit.fsu.edu) from 10,000 bootstrap pseudoreplicates. Only values >70% (considered significant) are shown. Different DOBV lineages are indicated by gray boxes. SANGV, Sangassou virus; HTNV, Hantaan virus; SEOV, Seoul virus; Saa, Saaremaa virus; Aa, *Apodemus agrarius*; Ap, *A. ponticus*; Af, *A. flavicollis*. WG, district Lüneburg, Lower Saxony (LS); Pe1 and Pe3, district Güstrow; H, district Nordvorpommern, K/A1, district Demmin, all Mecklenburg-Western Pomerania (MWP); To and Ka, district Ostprignitz-Ruppin, Brandenburg (BB). Before tree construction, automated screening for recombination between the S segment sequences was performed using program RDP3 (*15*), which used 6 recombination detection programs: Bootscan, Chimeric, GENECONV, MaxChi, RDP, and SiScan with their default parameters. No putative recombinant regions could be conclusively detected by >3 programs and subsequently verified by phylogenetic trees.

An initial screening by a large (L) segment–specific nested reverse transcription–PCR (RT-PCR) (*3*) of 67 lung samples, representing all seroreactive (n = 20) and equivocal (n = 6) as well as 36 selected seronegative and 5 serologically not-analyzed animals, showed a 390-nt amplification product for 21 samples representing 16 seroreactive, 4 seronegative, and 1 serologically not-investigated animals (Technical Appendix). To enable a comparison with the only available DOBV sequence from Germany (H169), an S segment portion of 559 nt was amplified by RT-PCR from 11 lung tissues (Technical Appendix). In the phylogenetic analyses, all sequences from Germany formed 1 well-supported (PUZZLE [www.tree-puzzle.de]) and bootstrap support values >90%) monophyletic group consisting of 2 clusters. The first cluster contained S segment sequences from district Güstrow (trapping sites Pe1 and Pe3), Lüneburg (trapping site WG), Nordvorpommern (trapping site H), and the previously published DOBV sequence from an HFRS patient from northern Germany (H169; [*8*]; Figure 2, panel A). A second cluster was formed by S segment sequences originating from districts Ostprignitz-Ruppin (trapping sites Ka, To) and Demmin (trapping site K/A1). Notably, the *A. flavicollis*–derived sequences from sites Pe1, H, Ka, and K/A1 clustered together or were completely identical with *A. agrarius*–derived sequences from the same or neighboring trapping sites, suggesting multiple spillover infections (Figure 2, panel A, Technical Appendix Table 1).

The sequences from Germany share a common ancestor with the DOBV-Aa sequences originating from Slovakia and Russia. Together, they form a monophyletic group (DOBV-Aa lineage) that is clearly separated from *A. flavicollis*–borne (DOBV-Af) and *A. ponticus*–borne (DOBV-Ap) sequences and from *A. agrarius*–borne Saaremaa virus sequences. Subsequent analysis of nucleotide sequences of the entire nucleocapsid (N) protein– and glycoprotein precursor (GPC)–encoding regions confirmed these findings (Figure 2, panel B; Technical Appendix). A pairwise comparison between nucleotide and amino acid sequences of the complete N and GPC open reading frames from the novel German DOBV strains showed divergences of 1.5%–8.8% (0.3%–1.4%) and 2.1%–8.3% (0.8%–1.8%), respectively (Technical Appendix Table 2). The highest identity values on the nucleotide and amino acid sequence level (91.2%–91.7% and 99%–99.7%) were found for an S segment sequence from Denmark (Lolland/1403; GenBank accession no. AJ616854; Technical Appendix). The nucleotide and amino acid sequence divergence to other DOBV sequences was much higher, reaching 10.1%–14.3% (1%–3.3%) and 12.6%–20.7% (2.9%–9.4%), respectively.

Morphologic species determination for all DOBV-seroreactive and RT-PCR–positive rodents was confirmed by a mitochondrial *cytochrome b* gene-specific PCR (*9*,*10*), sequence determination, and comparison with available GenBank sequences from *A. agrarius* and *A. flavicollis* (Technical Appendix Table 1).

## Conclusions

Based on a large panel of the entire N- and GPC-encoding DOBV sequences, we report direct molecular evidence that DOBV in Germany is represented by a genetic lineage associated with *A. agrarius* (DOBV-Aa). In contrast, we found no evidence for the occurrence of DOBV-Af in *A. flavicollis* or other *Apodemus* species from Germany. Consistent with the geographic distribution of *A. agrarius* (*11*) and the report of human DOBV disease exclusively in northern and northeastern Germany, this finding may confirm DOBV-Aa as the sole causative agent of DOBV infections in Germany (*4*; Robert Koch-Institut, SurvStat, www.rki.de).

Previously *A. agrarius*–associated Saaremaa virus was experimentally shown to be able to infect *A. agrarius* and *A. flavicollis* mice (*12*). We report multiple natural spillover infections of *A. flavicollis* by a DOBV strain originally hosted by *A. agrarius*. The observed spillover infections represent a crucial prerequisite for genetic reassortment. This observation is in contrast to other reports from Slovenia and Slovakia where, although *A. agrarius* and *A. flavicollis* are occurring sympatrically, *A. flavicollis* is exclusively carrying the DOBV-Af and *A. agrarius* the DOBV-Aa lineage (*4*,*13*). In contrast to our observations, single DOBV-Af spillover infections of *A. sylvaticus* and *Mus musculus* have been reported previously (*14*).

The phylogenetic analyses demonstrated 2 well-separated clusters within the DOBV-Aa lineage. These rodent-derived DOBV sequences in Germany represent a major contribution to the DOBV genomics and phylogenetics. Future investigations should help to identify specific features of these DOBV-Aa strains resulting in its frequent spillover to *A. flavicollis* and to prove a putative adaptation of DOBV-Aa on *A. flavicollis* after spillover, as well as possible reassortment processes.

## Supplementary Material

Technical Appendix Summary of the serologic and reverse transcription-PCR (RT-PCR) investigations of all serologically and/or RT-PCR-positive Apodemus mice*
