# Supplementary material for: Dobrava-Belgrade Virus Spillover Infections, Germany
Source: Emerg Infect Dis. 2009 Dec;15(12):2017–20. doi: 10.3201/eid1512.090923 (PMC3044545; doi:10.3201/eid1512.090923)
Supplement: Technical Appendix — Summary of the serologic and reverse transcription-PCR (RT-PCR) investigations of all serologically and/or RT-PCR-positive Apodemus mice* [file 09-0923_Techapp-s1.pdf]

Dobrava-Belgrade Virus Spillover Infections, Germany

Technical Appendix

Table 1. Summary of the serologic and reverse transcription-PCR (RT-PCR) investigations of all serologically and/or RT-PCR-positive *Apodemus* mice*

Federal state and district	Trapping site	Rodent species (sex)	Rodent no.	DOBV (Slo)-IgG-ELISA†	FRNT‡ DOBV-Aa (SK)	FRNT‡ DOBV-Af (Slo)	Nested RT-PCR L segment	Obtained sequences DOBV S segment	Obtained sequences DOBV M segment	Obtained sequences *cyt b* (partial)
Lower Saxony										
Lüneburg	WG	Aa (F)	GER/08/ 124/Aa	++	**80**	20	Pos	Partial (nt 1-934); identical to GQ205407	ND	GQ260183
		Aa (M)	GER/08/ 118/Aa	+++	40	40	Pos	GQ205407 Complete	GQ205412 complete	GQ260182
		Aa (F)	GER/08/ 125/Aa	+++	ND	ND	pos	Partial (nt 1-934); identical to GQ205407	ND	GQ260184
Mecklenburg-Western Pomerania										
Demmin	K/A1	Aa (M)	GER/07/ 1064/Aa	+++	**40**	20	pos	GQ205404 complete	ND	GQ260176
		Af (M)	GER/07/ 1058/Af	++	**640**	80	pos	Partial (nt 219-1675); identical to GQ205404	ND	GQ260175
		Aa (F)	GER/07/ 1081/Aa	++	ND	ND	pos	ND	ND	GQ260177
		Aa (M)	GER/07/ 1029/Aa	neg	ND	ND	pos	ND	ND	GQ421162
		Aa (F)	GER/07/ 1027/Aa	ND	ND	ND	pos	ND	ND	GQ421163
		Af (F)	GER/07/ 992/Af	neg	ND	ND	pos	ND	ND	GQ421166
Nordvor-pommern	H	Aa (F)	GER/08/ 83/Aa	++	**80**	40	pos	ND	ND	GQ260180
		Aa (F)	GER/08/ 79/Aa	+	ND	ND	pos	ND	ND	GQ260178
		Aa (M)	GER/08/ 80/Aa	+	ND	ND	pos	ND	ND	GQ260179
		Aa (M)	GER/08/ 84/Aa	+	ND	ND	pos	ND	ND	GQ260181
		Af (M)	GER/08/ 131/Af	+	**10**	<10	pos	GQ205408 complete	GQ205413 complete	GQ260185
		Aa (M)	GER/08/ 82/Aa	neg	ND	ND	pos	ND	ND	GQ421164
		Af	GER/08/	neg	ND	ND	pos	ND	ND	GQ421165

		(M)	133/Af							
Güstrow	Pe3	Aa (M)	GER/07/ 293/Aa	+++	**640**	80	pos	GQ205401 complete	GQ205409 complete	GQ260171
		Aa (F)	GER/07/ 634/Aa	+	ND	ND	neg	ND	ND	GQ260174
		Aa (M)	GER/07/ 372/Aa	+	ND	ND	neg	ND	ND	GQ260172
	Pe1	Af (F)	GER/07/ 607/Af	++	<10	<10	pos	GQ205402 complete	GQ205410 complete	GQ260186
		Aa (F)	GER/07/ 424/Aa	++	ND	ND	pos	GQ205403 complete	ND	GQ260173
Brandenburg										
Ostprignitz-Ruppin	To	Aa (M)	GER/05/ 239/Aa	+++	ND	ND	pos	GQ205405 complete	ND	GQ260167
		Aa (M)	GER/06/ 44/Aa	+	ND	ND	neg	ND	ND	GQ260169
		Aa (F)	GER/06/ 49/Aa	+	ND	ND	neg	ND	ND	GQ260170
	Ka	Af (M)	GER/05/ 477/Af	+	ND	ND	pos	GQ205406 complete	GQ205411 complete	GQ260168

*FRNT, focus reduction neutralization test; DOBV, *Dobrava-Belgrade virus*; Aa, *Apodemus agrarius*; Af, *A. flavicollis*; Slo, strain Slovenia; SK, strain Slovakia; *cyt b, cytochrome b*; ND, not done; nt, nucleotide; neg, negative; pos, positive.
†Optical density values: +++, ≥2.0; ++, 1.9-1.0; +, 0.9-0.2; neg, < lower cut-off (in average 0.041).
‡Endpoint titers.

Table 2. Pairwise nucleotide and amino acid sequence divergence between the entire N- and GPC-encoding DOBV S- and M-segment sequences originating from Germany to those from other regions in Europe*

		Country	% identity with strain												
Segment and strain	Species	(site)	1	2	3	4	5	6	7	8	9	10	11	12	13
S segment ORF															
1. GER/07/293	Aa	GER/Pe3	–	98.5	91.2	98.2	95.1	89.1	87.4	87.2	87.1	87.3	89.0	85.9	91.7
2. GER/07/607	Af	GER/Pe1	99.7	–	91.8	98.0	95.3	88.7	86.9	87.1	87	87.1	89.3	85.7	91.2
3. GER/05/477	Af	GER/Ka	98.8	99.0	–	91.2	91.9	88.9	87.5	86.5	86.8	86.0	89.9	86.7	91.4
4. GER/08/118	Aa	GER/WG	99.3	99.5	98.6	–	94.7	88.8	87.1	87.1	87	87.2	88.6	85.7	91.2
5. GER/08/131	Af	GER/H	99.5	99.7	98.8	99.3	–	89.3	87.4	88	88	88.1	89.6	87.5	91.4
6. SK/Aa	Aa	SVK	98.8	99.0	98.6	98.6	98.8	–	86.4	86.4	85.2	84.6	91.3	86.5	89.4
7. Saa/160V	Aa	EST	96.9	97.2	97.6	96.7	97.4	96.7	–	87.7	87.6	87.4	87.1	84.8	89.7
8. Slo/Af	Af	SVN	97.6	97.9	97.4	97.4	97.6	97.4	96.9	–	96	95.1	87.5	88.2	87.5
9. AP/Af19	Af	GRC	98.3	98.6	98.1	98.1	98.3	98.1	97.2	99.3	–	96.4	87.1	87.6	87.1
10. Esl400/Af	Af	SVK	98.1	98.3	97.9	97.9	98.1	97.9	96.9	99	99.7	–	87.1	87.4	86.6
11. Lipetsk/Aa	Aa	RUS	98.1	98.3	97.9	98.3	98.1	98.8	96.0	96.7	97.4	97.2	–	86.8	89.3
12. Sochi/Ap	Ap	RUS	97.6	97.9	97.4	97.4	98.1	97.4	96.2	97.6	97.9	97.6	96.7	–	86.8
13. Lolland/Aa1403	Aa	DNK	99.3	99.5	99.0	99.0	99.7	99.0	97.6	97.9	98.6	98.3	98.3	98.3	—
M segment ORF															
1. GER/07/293	Aa	GER/Pe3	–	97.9	91.7	97.5	93.2	87.0	86.9	82.8	82.1	82.6	86.7	79.3	–
2. GER/07/607	Af	GER/Pe1	99.1	–	91.9	97.9	93.5	87.4	87.2	83.4	82.4	83.2	87.1	79.5	–
3. GER/05/477	Af	GER/Ka	98.3	98.6	–	92.0	91.7	86.5	87.0	83.3	82.9	83.3	87.2	80.1	–
4. GER/08/118	Aa	GER/WG	98.7	99.2	98.4	–	93.0	86.9	87.4	83.3	82.5	83.0	86.9	79.7	–
5. GER/08/131	Af	GER/H	98.2	98.6	98.8	98.4	–	86.6	87.0	83.1	82.3	83.5	87.3	80	–
6. SK/Aa	Aa	SVK	96.0	96.4	96.2	96.2	96.4	–	87.1	82.5	82.3	82.5	87.6	79.5	–
7. Saa/160V	Aa	EST	95.7	96.0	95.7	95.8	95.9	95.8	–	82.2	82.4	82.1	86.4	79.1	–
8. Slo/Af	Af	SVN	93.6	94.0	94.1	93.8	94.0	93.6	94.1	–	93.7	92.8	83.5	80.4	–
9. AP/Af19	Af	GRC	93.7	94.1	94.2	93.9	94.0	93.6	94.1	98.6	–	93.2	83.2	80.4	–
10. Esl400/Af	Af	SVK	94.0	94.4	94.5	94.1	94.3	94.0	94.3	98.9	99.0	–	83.6	80.8	–
11. Lipetsk/Aa	Aa	RUS	96.7	97.1	96.7	97.0	96.6	97.0	96.2	94.0	94.1	94.4	–	80.6	–
12. Sochi/Ap	Ap	RUS	90.6	91.0	90.9	90.7	90.8	90.5	90.3	93.3	93.3	93.8	91.4	–	–

*Values above the diagonal are nucleotide sequence differences and values below the diagonal are amino acid differences. ORF, open reading frame.

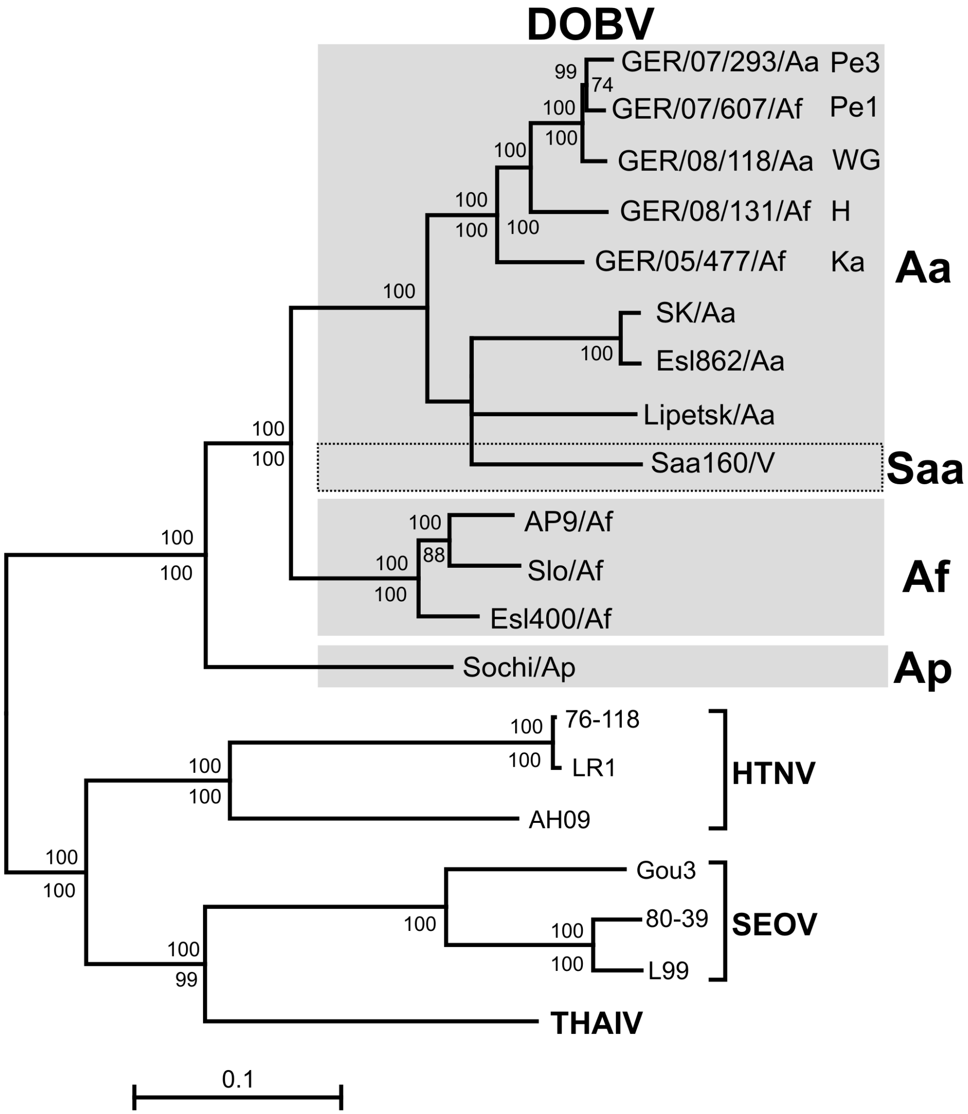

Figure. Maximum-likelihood (ML) phylogenetic tree of Dobrava-Belgrade virus (DOBV) based on complete glycoprotein precursor coding nucleotide sequences (M-segment open rading frame) of 3,405 nt. The ML tree (Tamura-Nei evolutionary model) was calculated using TREE-PUZZLE package (www.tree-puzzle.de). Scale bar indicates an evolutionary distance of 0.1 substitutions per position in the sequence. Values above the branches represent PUZZLE support values. Values below the branches are the bootstrap values of the corresponding neighbor-joining tree (Tamura-Nei evolutionary model) calculated with the PAUP* software from 10,000 bootstrap pseudoreplicates. Phylogenetic trees with all evolutionary models available in TREE-PUZZLE were constructed and compared using statistical tests implemented in TREE-PUZZLE (Kishino-Hasegawa test, Shimodaira-Hasegawa test, and Expected Likelihood Weight). In most cases, they were not significantly different and therefore only trees with Tamura-Nei evolutionary model, which showed highest PUZZLE and bootstrap support values, are shown. Different DOBV lineages are indicated by gray boxes. HTNV, *Hantaan virus*; SEOV, *Seoul virus*; THAIV, *Thailand virus*; Saa, Saaremaa virus; Aa, *Apodemus agrarius*; Ap, *A. ponticus*; Af, *A. flavicollis*. WG, district Lüneburg, Lower Saxony (LS); Pe1 and Pe3, district Güstrow; H, district Nordvorpommern, K/A1, district Demmin, all Mecklenburg-Western Pomerania (MWP); To and Ka, district Ostprignitz-Ruppin, Brandenburg (BB). By automated screening for recombination between the M-segment sequences using program RDP3 (15) no putative recombinant regions could be detected by $\geq$3 programs and subsequently verified by phylogenetic trees.
